# Supplementary material for: Modeling glioblastoma heterogeneity as a dynamic network of cell states
Source: Mol Syst Biol. 2021 Sep 16;17(9):e10105. doi: 10.15252/msb.202010105 (PMC8444284; doi:10.15252/msb.202010105)
Supplement: Supplementary file 5 — Source Data for Figure 3 [file MSB-17-e10105-s001.zip › Figure3A_sourcedata/GSEA_3065/nosigdb_state2.GseaPreranked.1622718419215/ANASTASSIOU_MULTICANCER_INVASIVENESS_PMID_22208948.html]

Details for gene set ANASTASSIOU\_MULTICANCER\_INVASIVENESS\_PMID\_22208948[GSEA]

|  || Dataset | state2 |
| Phenotype | NoPhenotypeAvailable |
| Upregulated in class | na\_neg |
| GeneSet | ANASTASSIOU\_MULTICANCER\_INVASIVENESS\_PMID\_22208948 |
| Enrichment Score (ES) | -0.7801179 |
| Normalized Enrichment Score (NES) | -2.3919196 |
| Nominal p-value | 0.0 |
| FDR q-value | 0.0 |
| FWER p-Value | 0.0 |
Table: GSEA Results Summary

  

Fig 1: Enrichment plot: ANASTASSIOU\_MULTICANCER\_INVASIVENESS\_PMID\_22208948      
 Profile of the Running ES Score & Positions of GeneSet Members on the Rank Ordered List

  

| PROBE | GENE SYMBOL | GENE\_TITLE | RANK IN GENE LIST | RANK METRIC SCORE | RUNNING ES | CORE ENRICHMENT || 1 | RAB31 |  |  | 1977 | 0.065 | -0.1940 | No |
| 2 | TNFAIP6 |  |  | 2720 | 0.041 | -0.2626 | No |
| 3 | RCN3 |  |  | 5254 | -0.004 | -0.5307 | No |
| 4 | NID2 |  |  | 5511 | -0.009 | -0.5558 | No |
| 5 | MMP11 |  |  | 6202 | -0.020 | -0.6242 | No |
| 6 | AEBP1 |  |  | 7162 | -0.041 | -0.7160 | No |
| 7 | TIMP3 |  |  | 7309 | -0.045 | -0.7205 | No |
| 8 | LOXL2 |  |  | 7871 | -0.062 | -0.7649 | Yes |
| 9 | NUAK1 |  |  | 7976 | -0.066 | -0.7596 | Yes |
| 10 | FBN1 |  |  | 8107 | -0.072 | -0.7558 | Yes |
| 11 | PLAU |  |  | 8131 | -0.073 | -0.7402 | Yes |
| 12 | THY1 |  |  | 8139 | -0.074 | -0.7228 | Yes |
| 13 | PRRX1 |  |  | 8427 | -0.091 | -0.7310 | Yes |
| 14 | COL11A1 |  |  | 8598 | -0.103 | -0.7237 | Yes |
| 15 | LGALS1 |  |  | 8680 | -0.110 | -0.7054 | Yes |
| 16 | PCOLCE |  |  | 8685 | -0.110 | -0.6787 | Yes |
| 17 | NTM |  |  | 8787 | -0.120 | -0.6600 | Yes |
| 18 | ADAM12 |  |  | 8799 | -0.122 | -0.6311 | Yes |
| 19 | TMEM158 |  |  | 8846 | -0.128 | -0.6044 | Yes |
| 20 | CDH11 |  |  | 8983 | -0.146 | -0.5830 | Yes |
| 21 | COL6A2 |  |  | 9116 | -0.170 | -0.5553 | Yes |
| 22 | COL5A1 |  |  | 9148 | -0.177 | -0.5151 | Yes |
| 23 | MMP2 |  |  | 9307 | -0.235 | -0.4740 | Yes |
| 24 | COL5A2 |  |  | 9316 | -0.244 | -0.4149 | Yes |
| 25 | FN1 |  |  | 9364 | -0.294 | -0.3475 | Yes |
| 26 | SPARC |  |  | 9384 | -0.332 | -0.2680 | Yes |
| 27 | VCAN |  |  | 9396 | -0.364 | -0.1798 | Yes |
| 28 | POSTN |  |  | 9437 | -0.750 | 0.0003 | Yes |
Table: GSEA details [plain text format]

  

Fig 2: ANASTASSIOU\_MULTICANCER\_INVASIVENESS\_PMID\_22208948: Random ES distribution      
 Gene set null distribution of ES for **ANASTASSIOU\_MULTICANCER\_INVASIVENESS\_PMID\_22208948**
